# Art and science: how musical training shapes the brain

**DOI:** 10.3389/fpsyg.2013.00713

**Published:** 2013-10-16

**Authors:** Karen Chan Barrett, Richard Ashley, Dana L. Strait, Nina Kraus

**Affiliations:** ^1^Auditory Neuroscience Laboratory, Department of Communication Science and Disorders, Northwestern UniversityEvanston, IL, USA; ^2^Program in Music Theory and Cognition, Bienen School of Music, Northwestern UniversityEvanston, IL, USA; ^3^Music Cognition Laboratory, Program in Music Theory and Cognition, Bienen School of Music, Northwestern UniversityEvanston, IL USA; ^4^Program in Cognitive Science, Northwestern UniversityEvanston, IL, USA; ^5^Institute for Neuroscience, Northwestern UniversityChicago, IL, USA; ^6^Department of Communication Sciences and Disorders, Northwestern UniversityEvanston, IL, USA; ^7^Department of Neurobiology & Physiology, Northwestern UniversityEvanston, IL, USA; ^8^Department of Otolaryngology, Northwestern UniversityEvanston, IL, USA

**Keywords:** neural plasticity, musical training, talent, cognition, brain

## Abstract

What makes a musician? In this review, we discuss innate and experience-dependent factors that mold the musician brain in addition to presenting new data in children that indicate that some neural enhancements in musicians unfold with continued training over development. We begin by addressing effects of training on musical expertise, presenting neural, perceptual, and cognitive evidence to support the claim that musicians are shaped by their musical training regimes. For example, many musician-advantages in the neural encoding of sound, auditory perception, and auditory-cognitive skills correlate with their extent of musical training, are not observed in young children just initiating musical training, and differ based on the type of training pursued. Even amidst innate characteristics that contribute to the biological building blocks that make up the musician, musicians demonstrate further training-related enhancements through extensive education and practice. We conclude by reviewing evidence from neurobiological and epigenetic approaches to frame biological markers of musicianship in the context of interactions between genetic and experience-related factors.

“I've never known a musician who regretted being one. Whatever deceptions life may have in store for you, music itself is not going to let you down.”

Virgil Thomson, composer

## Introduction

To be a musician is to be a consummate multi-tasker. Music performance requires facility in sensory and cognitive domains, combining skills in auditory perception, kinesthetic control, visual perception, pattern recognition, and memory. Because of its cognitive demands and the coupling required across sensory systems, musical training has provided a fruitful model for studying plastic changes in the brain and behavior that occur through short- and long-term training (e.g., Rauschecker, 2001; Münte et al., 2002; Stewart, 2008; Habib and Besson, 2009; Wan and Schlaug, 2010; Herholz and Zatorre, 2012; Strait and Kraus, 2013). In the case of professional musicians, training occurs over a lifetime, often commencing at a young age. Basic music skills can also be taught to novice participants in an experimental setting, allowing the examination of short-term training effects. Despite a wealth of studies that have investigated biological markers of musical training, we cannot yet answer a fundamental question: are musicians born or made?

In this review we interpret new and previously established findings according to the argument that many of musicians' biological distinctions develop in combination with or as a result of rigorous musical training rather than intrinsic advantages alone. Considering evidence from longitudinal and cross-sectional studies, we summarize support for (1) anatomical differences in the brains of musicians, (2) musician advantages in aspects of the neural encoding of sound, and (3) cognitive and perceptual advantages that relate to extent of musical training.

A gold standard experimental model for learning/training studies is random assignment to an experimental group with two control groups: an active control and a passive control, with the training groups undergoing systematic, consistent regimens. Without random assignment, pre-existing differences in motivation or ability that caused some people to pursue music in the first place may impact outcomes. Not using an active control group leaves open the possibility that trained individuals are improving relative to controls simply due to the extra attention they are receiving from instructors. In practice, few if any fully controlled studies of musical training can hew to these stringent criteria because of challenges inherent in assessment of training in a real-world setting.

It can, for example, be very difficult to conduct a study on the effects of musical training with random assignment, as subjects who are interested in musical training may be unwilling to postpone the start of their training until after the completion of the study. Furthermore, finding an active control training regimen that matches musical training in intensity and motivation can be logistically difficult. There is often, therefore, a tradeoff between the ecological validity of the musical instruction and the extent to which study designs can meet these criteria. Using training programs developed by the experimenter can enable more rigorously controlled studies but the results of such studies will be somewhat difficult to generalize to real-world music learning environments. On the other hand, by studying existing programs that have been demonstratively successful in teaching children musical skills, researchers can maximize the applicability of their research to educators, at the cost of certain limitations in study design. Nevertheless, the field of neuroscience is well acquainted with physiological outcomes of long- and short-term training and sensory enrichment, such as that which occurs through musical training. The consideration of musician enhancements as a result of practice, at least in part, yields insights into how the commitment to musical practice shapes human biology. Understanding the important role of predispositions, we discuss how experience and innate factors may interact to shape the brains and abilities of musicians. Taken together, the body of knowledge about the effects of musical training has been accumulated through a broad variety of study designs which, as a whole, support the notion that musical training can enhance neural, cognitive and communication function.

## Auditory functional and anatomical differences in the brains of musicians

Neural plasticity, consisting of changes in brain function or structure that affect behavior or cognition, underlies development, learning, rehabilitation from trauma, and skill refinement. The consistent regimen that musicians undertake to master an instrument relies on the brain's ability to learn—enabled by neural plasticity. Musical performance, whether via a physical instrument or the voice itself, involves disciplined muscle control, using body movements to produce carefully crafted sounds.

It is not surprising that anatomical differences have been found between musicians' and non-musicians' auditory and motor cortices and the neural connectivity linking these areas. Adult instrumental musicians, for example, have more gray matter in somatosensory, premotor, superior parietal, and inferior temporal areas of the cortex and these enlargements correlate with their levels of expertise (Gaser and Schlaug, 2003). Musicians also have larger cerebellar volume, with the extent of this greater volume correlating with the lifelong intensity of musical practice, which has been proposed to be due to the role of the cerebellum in motor and cognitive skill learning (Hutchinson et al., 2003). The degree of musical achievement likewise correlates with more gray matter volume in Heschl's gyrus, an area of the auditory cortex linked to abilities in pitch discrimination and detecting tonal patterns (Schneider et al., 2002). In a study investigating non-musicians, amateur musicians, and expert musicians, increased musical expertise correlated with gray matter density in areas involved with higher order cognitive processing and auditory processing (James et al., 2013). Interestingly, increased expertise was also linked to a decrease in gray matter density in areas related to sensorimotor function, proposed to be due to an increased automatization of motor skills or higher motor efficiency (James et al., 2013). Taken together, these findings imply that changes in the brain's auditory and motor areas relate to active music-making.

Similar structural brain distinctions have been found in child musicians in the early stages of honing their musical skills. Schlaug et al. (2005) tracked 5–7 year old children as they progressed with their musical studies. While they observed no preexisting cognitive, musical, motor, or structural brain differences between the subsequently musically trained and control groups, children who studied music for 12 months developed enhanced activation of the bilateral temporal lobes and superior temporal gyri during rhythmic and melodic discrimination tasks. After 15 months of piano lessons, children further showed training-related changes in the motor cortex, the corpus callosum, and the right Heschl's gyrus compared to controls (Hyde et al., 2009; Schlaug et al., 2009b), the same areas of the brain that are enhanced in adult musicians (see above). Since the children who took part in this study chose to participate in music lessons and were not randomly assigned, we cannot evaluate the contribution of predispositions not captured by initial group comparisons. However, from these longitudinal results we can conclude that structural neural changes unfold with music learning in children amenable to undergoing lessons early in life, when the brain is most flexible and dynamic. To explore this matter, a study of how 15 months of piano lessons impacts brain development in children not drawn to learning an instrument would be needed.

### Neural connectivity

Much of the research on music and neural plasticity has focused on gray matter volume in cortex with musical training, which may reflect increased neuronal or synaptic count; increased gray matter may drive the growth of new dendrites and the disinhibition or inhibition of pre-existing synaptic connections (for further discussion see Münte et al., 2002). To better understand neural connectivity in the brains of musicians, researchers have investigated white matter differences, reflecting volumetric differences in the nerve fibers that underscore neural connectivity. Musicians have a larger corpus callosum, the fiber tract underlying most interhemispheric communication, with musicians who started training at an earlier age having a larger corpus callosum compared to musicians who started later (Schlaug et al., 1995; Wan and Schlaug, 2010). Musicians' larger corpus callosum volume may reflect decreased interhemispheric inhibition (Ridding et al., 2000) and more communication between the two hemispheres.

White matter tracts are thought to continue developing until the age of 30 and the volume of certain fiber tracts (e.g., frontal and left temparoparietal tracts) has been linked to cognitive skills (Nagy et al., 2004; see also Schmithorst and Wilke, 2002; Bengtsson et al., 2005). It is interesting therefore to consider white matter characteristics in musicians, whose training peaks prior to the maturation of neuronal connectivity and may interact with their development. Pianists, for example, have more voluminous fiber tracts in the isthmus extending into the upper splenium (i.e., tracts that connect auditory regions) and in the frontal lobe (tracts supportive of motor sequencing, especially independent finger movements) than non-musicians. Musicians who began playing earlier in childhood demonstrate even greater enhancements in white matter volume, indicating that more extensive white matter plasticity may occur when training initiates earlier in development (Bengtsson et al., 2005). Musicians likewise have larger volume in the arcuate fasciculus, a fiber tract connecting motor and auditory regions (Halwani et al., 2011). The effect of music training on auditory-motor connectivity may yield clinical benefits: melodic intonation therapy, a song-like intonation-based speech therapy, increases arcuate fasciculus volume in aphasic stroke patients with concurrent improvements in speech production (Schlaug et al., 2008, 2009a).

Still, simply having greater white matter volume does not indicate that enhancements are of functional use to musicians, nor that they stem solely from musical training. Work on the biology of beat perception, however, indicates that some of musicians' enhancements may stem from regular interactions with musical sound. Auditory and motor regions comprising of a cortical-subcortical network including the putamen, supplementary motor area, and premotor cortex is generally activated in human listeners when perceiving beats, with increased functional coupling between auditory and motor areas observed in musicians (Grahn and Rowe, 2009). Musicians' increased audio-motor co-activation during their consistent interactions with musical sound may induce structural changes in the white matter tracts bridging auditory and motor sites. This may account for musicians' more efficient audiomotor learning, a skill that allows them to not only perform music (see Schlaug et al., 2005; Watanabe et al., 2007; Forgeard et al., 2008) but that translates to other tasks such as musicians' ability to more accurately pronounce foreign languages (Milovanov et al., 2010) or have superior spatial tactile acuity (Ragert et al., 2004).

### Maladaptive plasticity

Volumetric brain differences among musicians compared to non-musicians cannot be considered beneficial to musicians unless such brain differences result in functional enhancements. Too much plasticity, in fact, can be harmful. Focal dystonia, a condition involving involuntary movements and muscle contractions, has long plagued musicians including the 19th-century composer/pianist Robert Schumann and current-day concert artists such as pianist Leon Fleisher and oboist Alex Klein. Also known as the “musician's cramp,” focal dystonia may result from maladaptive plasticity: fMRI scans of 5 dystonic guitarists showed abnormal recruitment of cortical areas involved in control of voluntary movement, exhibiting significantly greater activation of the contralateral primary sensorimotor cortex and underactivation of premotor areas (Pujol et al., 2000). It has been proposed that the disorganization of sensory inputs induced by over-use lead to poorly differentiated motor representations, providing the underlying mechanism for faulty motor control in dystonic instrumentalists (Pujol et al., 2000 and discussion in Pascual-Leone, 2001). Although speculative, the pattern of neural findings (overdevelopment of sensory function with diminished cognitive control) suggests that sensory automaticity from repeated sound-to-meaning associations (Baldeweg, 2006; Nelken and Ulanovsky, 2007; Ahissar et al., 2009; Conway et al., 2009; Chandrasekaran and Kraus, 2010) fails to develop or becomes disrupted.

While we have made great headway toward defining maladaptive outcomes of music-related neuroplasticity with regard to motor function, maladaptive effects in musicians have not been defined in other domains. Future research might probe musicians' neural profiles to determine whether their extensive functional and anatomical enhancements develop to the detriment of others.

## Experience-related contributions to the musician's auditory system

Musical training relates to functional advantages for processing discrete features of sound (for review, see Kraus and Chandrasekaran, 2010; Strait and Kraus, 2013) using the auditory brainstem response to complex sounds (cABR) as a metric (for further definition of the cABR see Skoe and Kraus, 2010). This work has revealed that musicians demonstrate faster and more robust auditory responses to sounds, ranging from music (Musacchia et al., 2007) to speech (Wong et al., 2007; Parbery-Clark et al., 2009a, 2012b; Bidelman et al., 2011; Strait et al., 2012b, 2013a) to emotionally communicative utterances (Strait et al., 2009). Children and adults undergoing musical training show more distinct neural encoding of stop consonants (Strait et al., 2013b) and less degradation of the neural response in the presence of background noise (Strait et al., 2013a, Figure 1). That children participating in school-based music programs are better at rhythmic tapping tasks (Slater et al., in press) may be reflected in more consistent cABRs, known to relate to better tapping ability (Tierney and Kraus, 2013). Musician brains also make greater use of acoustic context: adult musicians demonstrate larger neural responses to a speech syllable when it is presented regularly than in a variable context (Parbery-Clark et al., 2011b). These results suggest that, through training, musicians may be able to better sense relationships between sounds. This claim is supported by musicians' better performance on novel language- (Shook et al., 2013) and tonal sequence-learning tasks (Francois and Schon, 2011; Schon and Francois, 2011; Skoe and Kraus, 2013). This ability to neutrally process discrete sound features and detect sound patterns may translate into skills that underlie language learning in addition to helping musicians make sense of musical phrases and form, a process that is of vital importance for the detection, organization, and understanding of musical patterns.

**Figure 1:**
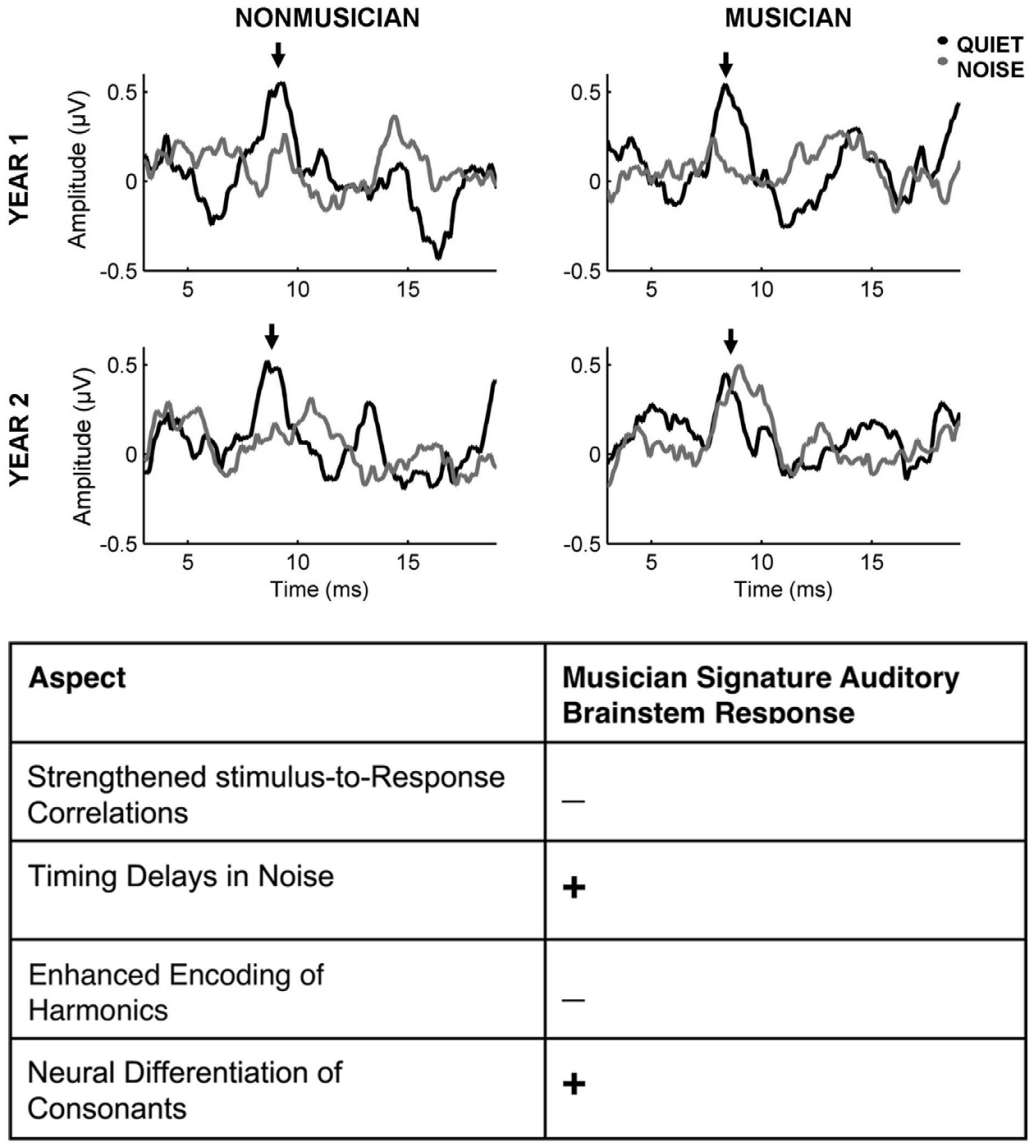
**Emerging neural enhancements in the encoding of sound in preschool-aged musicians with continued musical training. Top**: Representative auditory brainstem onset responses to /da/ in quiet and /da/ in noise for musician and non-musician participants who were tested initially and returned for testing after one year of continued musical training or alternative enrichment activities. Musicians have more resilient responses in noise after an additional year of musical training and development compared to non-musician counterparts (adapted from Strait et al., 2013b). **Bottom**: Preschool-aged musicians do not yet demonstrate the full “musician signature” enhancements seen in adults but aspects of enhanced neural encoding are beginning to emerge, as indicated by a + (Strait et al., 2013b, 2012b for review see Strait and Kraus, 2013).

**Figure 2:**
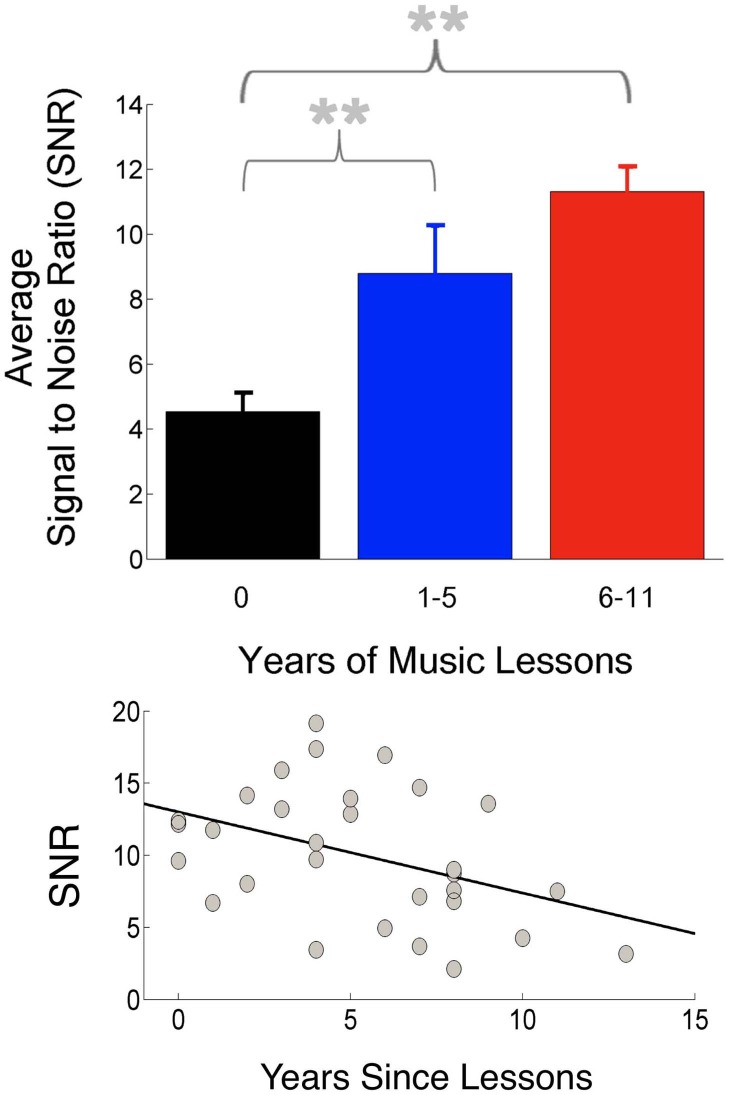
**Musical practice during childhood leads to more robust neural responses in adulthood. Top**: Participants were divided into three groups based on amount of musical practice. The participant group with no musical training (black) had the smallest amplitudes (highest SNR) in the frequency-following subcortical response compared to groups with more 1–5 years of musical training (blue) and 6–11 years of training (red). **Bottom**: Response magnitudes correlated with how recently musical training had ceased (adapted from Skoe and Kraus, 2012). ^**^p < 0.01.

Although innate predispositions likely guide the pursuit of music training and may differentiate musicians from non-musicians even before training begins, one cannot ignore the importance of interactive and consistent engagements with sound in the musician's world. It would be surprising were it not the case that musicians' auditory systems become adept at processing acoustic information, even beyond potential innate predispositions, given the profound impact of experience on the nervous system. This may account for the frequent relationships observed between the degree of musicians' neural enhancements with their extent of music training. Further support for a training-related component to musicians' neural enhancements comes from cohorts of musicians who trained as children but stopped instrumental training later in adulthood: neural responses to sound correlate with how recently training had ceased (Figure 2), suggesting that even a limited period of music lessons in the past changes how the brain later encodes sound (Skoe and Kraus, 2012; see also White-Schwoch et al., in press), consistent with animal work indicating that auditory training in early life can lead to benefits in task performance in adulthood (Sarro and Sanes, 2011). This work suggests that predispositions toward persevering with music training are not necessary for effects to be observed. Furthermore, this work adds to the evidence that musical training need not be pursued to a professional level for participants to reap neural benefits.

Support for a training-related component to musicians' enhanced neural response to sound may be gleaned from longitudinal work: preschoolers undergoing music lessons demonstrate more precise neural timing to speech in the presence of noise following one year of continued development and musical training whereas preschoolers engaged in non-musical activities show no measurable change (Strait et al., 2013b). Again, this longitudinal approach was conducted in children without random assignment and musicians enrolled in the study after the initiation of their training programs. Moving forward, longitudinal studies with random assignment, assessing auditory function pre- and post-training onset, should more fully delineate which aspects of auditory processing change with limited music experience, whether sensitive developmental periods constrain musical training's biological impact, and how much experience is required to bring about lasting neural effects.

**Figure 3:**
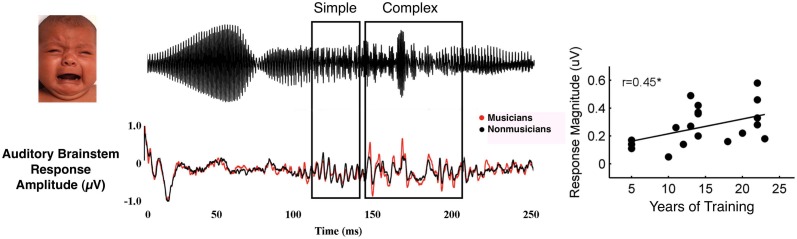
**Musician subjects demonstrate enhanced subcortical encoding (bottom waveform) to the complex portion of an emotional communication sound—a baby's cry (top waveform, see boxes dividing stimulus into simple and complex portions)**. Subcortical response magnitude in response to the complex portion correlates with years of musical training (adapted from Strait et al., 2009).

It is important to note that musical training does not result in an overall gain effect for auditory processing, with larger and faster responses occurring to all aspects of sound stimuli. For example, musicians have enhanced subcortical brain responses at distnctive times in the life span and these are limited to specific components of the response (Skoe and Kraus, 2013). Thus, training is associated with selective enhancements that may promote sensitivity to the most behaviorally relevant or acoustically complex aspects of sound or sound context. For example, when listening to an infant's unhappy cry, musicians have enhanced neural representation of the most acoustically complex portion of the stimulus (Figure 3; Strait et al., 2009). When listening to harmonic intervals, adult musicians demonstrate larger neural representation of the upper tones, the voice which often carries the melody in Western classical music (Lee et al., 2009), furthering work that has found enhanced cortical responses to the upper line or note in musical stimuli (Fujioka et al., 2005, 2008; Marie and Trainor, 2013; Butler et al., 2013). This neural evidence suggests that musical training may cause musicians to inherently attend to particular acoustic elements, potentially accounting for their skill extracting the most relevant and task-salient elements of a complex soundscape.

Cases where neural enhancements do not correlate with years of musical experience provide evidence contrary to the narrative that experience leads to enhancement; this evidence must be thoroughly considered. As with child musicians, young adult musicians have more robust neural representation of speech in the presence of noise (Parbery-Clark et al., 2009a) and better discrimination of stop consonants (Parbery-Clark et al., 2012b), but these enhancements do not correlate with extent of musical training. In older adults where aging has been associated with neural deficits, results have shown that musical experience mitigates aging's deleterious effects on neural encoding of speech (Parbery-Clark et al., 2012a), but these neural enhancements do not correlate with years of musical experience either. We must ask, why do certain neural enhancements in musicians correlate with extent of training in younger but not older populations? It is possible that some aspects of the neural encoding of sound are guided more by genetic factors and less by experience than others, or that a ceiling effect is reached according to which additional years of training cease to evoke further neural enhancements (for discussion see also Strait and Kraus, 2013). It is also possible that in older populations age-related declines may be counteracting the cumulative effects of musical experience.

In summary, musicians demonstrate enhancements in encoding fundamental aspects of sound when it comes to (1) complex sounds (e.g., communicative sounds, speech stimuli with pitch contours, musical intervals, acoustically similar stop consonants) and (2) complex acoustic environments (e.g., making sense of context), many of which increase with increased years of training. Given the rich soundscape that inundates the nervous system when playing music, it is unsurprising that musicians would develop auditory functional enhancements. However, we do not consider the demonstration of correlation the same as proof of causation and acknowledge the cautions of those who see a much larger role for genetics in addition to training. While cross-sectional studies comparing musicians and non-musicians are illuminating, longitudinal studies will more directly elucidate advantages with musical training and the time course over which they unfold.

### Tuned to timbre: instrument-specific encoding

**Figure 4:**
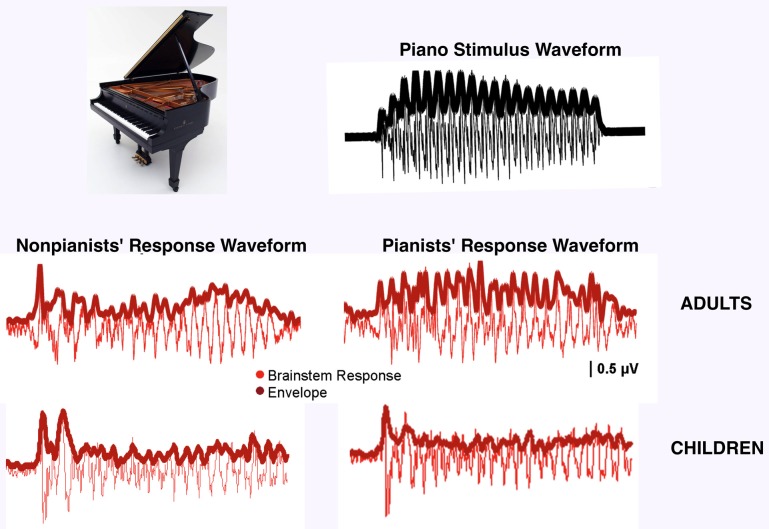
**Timbre specialization among musicians**. Adult pianists' auditory brainstem responses more closely reflect the amplitude envelope of the piano stimulus than non-pianists' (*F* = 6.97, *p* < 0.01), demonstrating preferential encoding for the sound of their instrument of practice (adapted from Strait et al., 2012a). Child pianists do not demonstrate preferential encoding of their instrument compared to non-pianists (*F* = 0.04, *p* = 0.85), perhaps indicating insufficient musical training to support specialization.

The studies considered above compared musicians to non-musicians but much can also be learned by looking within a group of musicians, observing how brain responses vary according to a specific type of musical training. Evidence for training-related neural plasticity is seen in musicians who have preferential neural responses to the sound of their instrument of practice; it is unlikely that these individuals were born with a neural predilection for their specific instrument. Musicians show preferential encoding for their own instrument in cortical-evoked potentials (Pantev et al., 2001), especially over right auditory cortex (Shahin et al., 2003). Musicians also have increased induced oscillatory gamma-band activity to their instrument of practice relative to others (Shahin et al., 2008). Neuroimaging reveals that musicians show enhanced neural activation when listening to musical compositions played by their own instrument in motor (precentral), auditory (superior temporal), syntactic (BA44) and executive (frontal) regions (Margulis et al., 2007). In addition to cortical specialization observed to the timbres of musicians' instruments, we have found similar specialization subcortically: when subdivided according to their instruments of practice (i.e., pianists and non-pianists), musicians demonstrate increased cABR fidelity to the sound of their own instrument relative to others (Figure 4; Strait et al., 2012a). We interpreted these data in the context of training-specific enhancements in musicians according to their musical practice histories. Non-peer reviewed work from our own group indicates that musicians' instrument-specific enhancements manifest behaviorally in enhanced online attention to the line played by their native instruments when listening to polyphonic music (Chan et al., 2010).

While the above experiments were performed on adults, cortical specialization for musical timbre has been observed even in children undergoing musical training. A study of non-musician and musician children prior to musical training showed no induced gamma-band activity to musical tones in either group. After half of these children underwent one year of piano training, however, these same children showed strengthened induced gamma-band activity in response to piano tones (Trainor et al., 2009). In fact, it was subsequently observed that infants simply exposed to music for ~160 min over the course of a week to a particular musical timbre demonstrate enhanced timbre-specific cortical responses (Trainor et al., 2011), supporting the interpretation that little instrumental exposure is necessary to induce cortical timbre specializations. To determine whether the expression of timbre-specific encoding within the auditory brainstem unfolds with similar rapidity, we collected cABRs to piano, bassoon and tuba tones in children who had undergone instrumental training for anywhere from 3 to 10 years, predicting that children would demonstrate preferential cABRs to their own instrument. This work revealed that even considerably musically trained children do not demonstrate the timbre-specific enhancements that are evident with comparably minimal musical training cortically (Figure 4; Chan et al., 2011). It is possible that cortical specialization to instrumental timbres precedes that measured subcortically. Cortical specialization may drive subcortical enhancements by means of the corticofugal system, fine-tuning subcortical auditory nuclei to the most behaviorally-relevant acoustic parameters for a given musician (Kraus and Chandrasekaran, 2010).

Musicians' strengthened encoding of their primary instrument may stem from hours of exposure and focused listening rather than innate predilections that would lead an individual to practice one instrument over another. Still, it possible that certain personalities—and associated underlying biologies—attract musicians to instruments; we could probe this question with thorough examination of personality and auditory evoked responses to musical timbres prior to giving children the opportunity to freely choose an instrument to study. At this time, the available data lead us to hypothesize that cortical and subcortical timbre specializations in adults and cortical timbre specializations in children provide support for use-related plasticity in musicians. It remains to be determined whether instrument-specific auditory processing in musicians lends to distinct neural advantages for processing non-musical sound, such as speech (e.g., are string players, who must constantly attend to and manipulate the pitch of their instrument, most sensitive to pitch deviations in speech?). Furthermore, what are the neural markers of playing multiple instruments or of having experience with other instruments through ensemble playing? Future experiments should investigate different types of training beyond simple divisions by instrument. By contrasting other types of pedagogical methods—e.g., classical vs. jazz training, score-based training vs. Suzuki aural methods—we may be able to determine whether various subcomponents of musical training differentially affect brain function. Such work could also yield outcomes for determining the optimal aspects of musical training for engendering neural benefits.

## Short-term plasticity: playing vs. listening to music

Again, the field of neuroscience is well acquainted with the effects of short-term training and sensory enrichment (Recanzone et al., 1993; Linkenhoker and Knudsen, 2002; Song et al., 2008; Anderson et al., 2013). One beneficial element of music as a training regimen lies in the fact that simple musical exercises can be taught to participants with no musical background. Experiments using this approach evidence the power of musical training's ability to induce rapid cognitive and neural benefits. Not surprisingly, immediate short-term training effects are seen in audio-motor areas as well as in auditory cortical-evoked responses.

In one approach, adult musicians were taught a keyboard exercise. The researchers observed that, with musical practice, neural sensorimotor representations of the finger muscles increased. Furthermore, following cessation of practicing the cortical maps returned to baseline (Pascual-Leone et al., 1995; Pascual-Leone, 2001). Whereas related evidence discussed in this article already was limited by approaches depending on group comparisons of non-musicians/musicians, these studies use a longitudinal approach with random group assignment. These studies thus provide evidence for musical training's contributions to structural aspects of sensorimotor cortices. Furthermore, this work suggests that, while sensorimotor cortices can by shaped by musical training, such plastic effects are not preserved when musical practice ceases. A related study reported parallel outcomes functionally when non-musicians learn keyboard tasks: piano training increases audio-motor co-activations in non-musicians after only 20 min of practice. This study further demonstrated that greater effects were observed with increased practice in that more extended and stable effects were observed after 5 weeks than with 20 min of practice (Bangert and Altenmuller, 2003).

Other studies have contrasted the effects of actively playing music to those of listening to music. Instrumental practice requires several interleaved processes—the translation of visual notation to motor movement, motor coordination between hands or between performers (see Loehr and Palmer, 2011), and auditory feedback once movements have been performed—that are not engaged when only listening to music. In a study examining cortical event-related potentials, non-musicians were either taught a piano sequence or trained to listen critically to music (Lappe et al., 2008). The group who practiced the sequences demonstrated larger mismatch negativity responses (MMNs) to unexpected tones after 2 weeks of training compared to the group who only listened to music (Lappe et al., 2008), thus showing a greater sensitivity to auditory anomalies. Similar effects have been seen when non-musicians were trained to either play rhythmic sequences or evaluate rhythmic accuracy in sequences. The non-musician group who played the piano sequence demonstrated larger MMN and P2 responses to rhythmic deviants after training compared to the matched non-musician group who critiqued rhythmic sequences (Lappe et al., 2011). These observations may relate to increased activity in frontoparietal motor areas when newly trained musicians listened to the music they had just learned, which has been attributed to the mirror neuron system (Lahav et al., 2007). Taken together, these results indicate that the practice of music—whether because of enhanced attention, physical engagement, or the combination of these—engenders neural changes related to auditory sensitivity that extend beyond those changes seen with auditory engagement through music listening. This work parallels experimental results from animal models where active training early in life leads to benefits in task performance in adulthood beyond those seen in a control group who only received passive exposure to these same sounds (Sarro and Sanes, 2011).

Plasticity with short-term musical training beyond those effects observed with short-term musical exposure has been similarly observed in babies who “play” music. Six-month old infants were randomly assigned either to an active music class consisting of group activities and playing simple instruments or to a passive music class where they listened to music but played no instruments. After six months of training, the infants in the active group demonstrated enhanced enculturation to Western tonal pitch structure, enhanced brain responses to musical tones, and increased social development relative to the listening group (Gerry et al., 2012; Trainor et al., 2012).

Together, these studies underline two important facts: (1) that active engagement with a musical instrument develops neural and behavioral enhancements that are greater than those seen from listening to music alone, and (2) that plastic changes occur in individuals without previous musical training. Thus, there is strong evidence for training-related plasticity in participants who did not independently gravitate toward musical training but were instead randomly selected to receive musical instruction. Furthermore, some changes in auditory and motor cortices occur on a rapid time scale, with adaptations seen as early as after 20 min of active playing. Future work might determine the time scales over which different neural enhancements unfold in musicians and during different developmental stages. Different musician-enhancements may be more or less malleable with short-term training approaches, occurring on different time scales or being predefined by intrinsic, inherited traits more than others. Given current evidence from short-term musical training approaches, future research should continue to disambiguate the distinctive biological effects of online, short-term, and long-term musical training as well as the effects of short-term musical training on other biological distinctions in musicians' such as cABRs.

## Cognitive and perceptual advantages induced by musical training

Musicians demonstrate better performance on a range of cognitive and perceptual tasks, some relating to the domain of music and some transferring to other domains, notably language. As detailed below, musicians have enhanced auditory attention and working memory, lending support to the idea that learning an instrument strengthens auditory abilities with collateral improvements in other domains (for review, see also Kraus et al., 2012).

Musical training has been associated with improved aspects of executive function and auditory attentional control, bringing about a wide array of cognitive benefits (Hannon and Trainor, 2007; Bialystok and DePape, 2009; Strait et al., 2010; Moreno et al., 2011; Strait and Kraus, 2011; Strait et al., 2012b). Musical training seems to hone auditory memory skills. Musicians have better auditory working memory (Chan et al., 1998; Jakobson et al., 2008; Parbery-Clark et al., 2009b, 2011a; Strait et al., 2012b, 2013a), potentially accounted for by musicians' increased activation of larger neuronal networks involved in cognitive control and sustained attention than non-musicians when confronted with difficult memory tasks (Gaab and Schlaug, 2003; Pallesen et al., 2010). Thus, it appears that performing music, creating sounds in the present while remembering their relation to past sounds, gives musicians a behavioral advantage in memory tasks that may be facilitated by different patterns of neural activation when engaging memory. Music's interactions with memory networks may account for why bards emphasized musical elements of language in the recitation of epic poems, which may have encouraged their memory consolidation (Bates, 1960; Kilgour et al., 2000; Jakobson et al., 2003, 2008). Music's relationship to memory may confer clinical benefits: it can “awaken” patients with dementia, allowing them to reconnect with reality through their memories of music (Cuddy and Duffin, 2005, see also Baird and Samson, 2009).

It has been proposed that musicians' better performance on cognitively demanding tasks reflects overall enhanced executive function (Bugos et al., 2007; Bialystok and DePape, 2009; Moreno et al., 2009) in conjunction with IQ (Schellenberg, 2004). In children, for example, the duration of music lessons positively correlates with IQ and academic ability (Schellenberg, 2006), although it is still unclear exactly what mediates this association (Schellenberg, 2011) and why these IQ effects are not always replicated (Brandler and Rammsayer, 2003; Moreno et al., 2009). However, the hypothesis that IQ or executive function drives such enhancements cannot account for musicians' strengthened performance on cognitively demanding auditory tasks beyond gains found in tasks performed in other domains (e.g., visual: Chan et al., 1998; Ho et al., 2003; Strait et al., 2010, 2012b; Parbery-Clark et al., 2011a).

Perceptual and cognitive enhancements with musical training have been evidenced more clearly through longitudinal studies. A group of 5–7 year olds about to start private music lessons were compared to a group of children not beginning instrumental training. The groups were initially matched with respect to neural anatomy (measured via MRI) as well as audio-cognitive, motor, and musical skills; however, after one year the musically-trained children demonstrated better auditory discrimination skills (Norton et al., 2005; Schlaug et al., 2005). Likewise, another longitudinal study tracked children after they were randomly assigned to complete either short-term computer-based musical training or short-term group painting lessons; the musical training group showed improved verbal intelligence and higher performance on an executive function task (go/no go) after only 20 days of training, with no changes observed according to these metrics in the painting group (Moreno et al., 2011).

Elderly populations may also derive cognitive enhancements from musical training (Bugos et al., 2007): elderly subjects who received individualized piano instruction for six months showed improved attention, working memory, and executive function (assessed via the Trail Making Test examining the ability to plan, execute, and modify a plan of action). Considering the extent of cognitive decline that accompanies aging, musical training may thus serve as a powerful cognitive rehabilitation technique in older adults. Finally, musicians' improved cognitive abilities relative to non-musicians may underlie their better hearing in noise (Parbery-Clark et al., 2009b; Strait et al., 2012b), auditory working memory, and pitch discrimination (although this advantage may be limited to frequencies typical in the range of music; see Schellenberg and Moreno, 2010), observed in musicians across the lifespan (Table 1)—including aging musicians (Parbery-Clark et al., 2011a, 2012a; Zendel and Alain, 2012).

**Table 1:** **Cognitive and perceptual skills correlate with musical experience in three different age-groups of musicians**.

**Behavioral measure**	**Older adults**	**Younger adults**	**School-aged children**
Speech-in-noise perception	ρ = −0.09	*r* = −0.58^**^	*r* = 0.63^***^
Auditory working memory	ρ = −0.14	*r* = 0.61^***^	*r* = −0.40^*^
Visual working memory	ρ = −0.09	ρ = 0.12	*r* = 0.20
Auditory attention	*r* = 0.11	*r* = −0.49^**^	*r* = −0.48^*^
Visual attention	*r* = 0.30	*r* = −0.30	*r* = 0.14
Frequency discrimination	*r* = 0.17	*r* = −0.40^*^	*r* = −0.38
Temporal acuity (Backward masking)	ρ = −0.03	*r* = −0.41^*^	*r* = −0.19

*Table summarizes Pearson's r or Spearman ρ correlation values between years of musical experience and behavioral measures. Bolded values indicate skills where musicians have behavioral advantages over non-musicians (Strait et al., 2010, 2012b; Parbery-Clark et al., 2011a). Skills that correlate with years of musical practice are generally seen in the auditory domain and are mostly seen in younger musicians*.

*** p < 0.001,

** p < 0.01,

* p < 0.05, ~p < 0.1.

## The combined roles of innate predispositions and experience-related factors

An obvious innate aspect of musical talent relates to the physicality required to master a particular instrument. For example, one could speculate how height conveys an advantage when playing a large instrument like the double bass and tidal volume (i.e., lung capacity) helps with playing a wind instrument. While proper training and technique can overcome physical limitations, it is advantageous to be born with the ideal physical abilities for a particular instrument. Less obvious, however, might be innate qualities related to temperament: differences in personality predict whether an individual will take music lessons and for how long they will engage in lessons. Among the Big 5 personality dimensions, openness to experience seems to have the highest power in predicting musical engagement, suggesting that future studies correlating abilities with musical training examine individual differences in personality (Corrigall et al., 2013). The ability to remain motivated and disciplined, which is a pre-requisite for successful musical training, may also be a function of innate makeup.

While innate factors that contribute to a musicians' pursuit of training surely exist, even innate predispositions can be shaped by environmental factors. For example, a higher musical capacity may be maximized or weakened over development due to access to or lack of training. This is demonstrated in absolute pitch, which seems to require both a genetic predisposition and early exposure to musical training (Baharloo et al., 1998; Levitin, 1994; Zatorre, 2003; Henthorn and Deutsch, 2007). Certain musical biases and dispositions may be rooted in innate predispositions (see Pond, 1981; Trehub, 2003) but through additional instruction, individuals hone the ability to artfully interpret music and convey it, creatively and expressively, to an audience (for discussion, see Williamon and Valentine, 2000). Finally, audiation, otherwise known as musical imagery or the “capacity to internalize musical sound and … ideas” (Keller, 2012), is thought to be a key component of musical aptitude. Although speculative, music educators have proposed that students are born with the potential to develop audiation skills but that the appropriate musical environment is necessary to maximize that potential (Gordon, 1999).

Effects of pleasure associated with music may provide another example of the potential for interactions between innate predispositions and experience. Music activates neural circuits involved in emotion and reward (Blood et al., 1999; Blood and Zatorre, 2001; Salimpoor and Zatorre, 2013; Salimpoor et al., 2013), with intensely pleasurable responses to music co-occurring with dopaminergic activity in the striatal system (Salimpoor et al., 2011). However, little is known about individual variability in music-induced emotional responses. It may be the case that individuals with innate attractions to musical training experience greater emotional rewards in its presence. Music students who receive the greatest emotional reward during music practice may be more likely to continue training, gaining increased biological benefits relative to students forced to continue participation by some external force. This would imply a feedback system in which a predisposition toward music is rewarded by a feeling of satisfaction, which in turn encourages a student to practice. Alternatively, depending on the individual, it is possible that since emotional engagement and motivation do not necessarily need to be pleasurable for learning to take place (Rutkowski and Weinberger, 2005; David et al., 2012), a temperament that is capable of tolerating frustration while persevering may also be more likely to continue with lessons to acquire musical skills (Ericsson et al., 1993; Ericsson and Lehmann, 1996).

Genetic and experience-related factors may weigh more heavily at different developmental time points. While the auditory system begins to develop in utero, a process that is mediated by genetic mechanisms (Clopton and Silverman, 1977; Taniguchi, 1981; Gordon, 2001; Tillein et al., 2012), this early development contrasts the later maturation of cortex, with the development of superficial layers extending into adulthood (Moore et al., 1995; Moore and Guan, 2001; Moore and Linthicum, 2007). Auditory input at key points throughout this developmental process, especially during early developmental years (Zhang et al., 2001), is necessary to refine how the auditory system makes use of basic encoding mechanisms for processing acoustic input (Gordon, 2001; Chang and Merzenich, 2003; Chang et al., 2005; Kral and Eggermont, 2007; Gordon et al., 2011). This developmental perspective may account for different neurobiological profiles observed in musicians who initiated training before or after early childhood (i.e., age seven; Strait et al., 2009; Steele et al., 2013).

Epigenetic mechanisms—factors that alter gene expression—can be influenced by environment and life experiences such that epigenetic variation may be a mechanism for experience-related developmental plasticity (for review, see Champagne and Curley, 2011; Champagne, 2013). These variations are induced by the presence of populations of proteins called transcription factors that work together to influence gene expression; the activity of different transcription factors can be facilitated or depressed due to enriched or deprived experiences, especially early in life. For example, the expression of N-Methyl-aspartate (NMDA) glutamate receptors has been implicated in increasing synaptic efficacy to bring about learning, with more effective learning taking place in animals with more NMDA receptors (for review, see Lau and Zukin, 2007). Auditory enrichment early in life through music exposure has been linked to increased NMDA receptor expression in the rat auditory cortex, paralleled by improved performance on auditory discrimination and learning and memory tasks (Tang et al., 1999; Xu et al., 2009; see also Cui et al., 2009). While music exposure's impact on NMDA receptor expression has not yet been compared to other forms of auditory enrichment, it is possible that early enrichment through musical training may cause an upregulation of proteins like NMDA receptors to promote synaptic efficacy and improve auditory learning abilities, in turn feeding back to promote musical development.

## Conclusions

This paper has presented support for training-related plasticity leading to neuroanatomical differences and improved neural encoding of sound. This paper has also considered the impact of musical training via a combination of genetics and experience.

It is clear that even among professional musicians there are inter-individual distinctions that lead to differences in performance capabilities. Still, one need not be a musical prodigy to gain benefits from musical training. Since musical aptitude exists on a wide spectrum, future research might continue to delineate what distinguishes biological and cognitive enhancements in professional, amateur, and novice musicians. Furthermore, we should continue to examine to what extent sensitive, or even critical, periods exist for different musical skills (e.g., Hannon and Trainor, 2007).

Continued research using longitudinal studies with random assignment, active controls, as well as insights gained from outcomes of community- and lab-based courses of training will continue to reveal effects of musical practice. Experimental investigation of individual differences among musicians may elucidate those abilities that result from innate predispositions. Future research may also clarify the cellular mechanisms underlying neural plasticity, including genetic markers underlying related abilities and their sensitivity to epigenetic modification.

### Conflict of interest statement

The authors declare that the research was conducted in the absence of any commercial or financial relationships that could be construed as a potential conflict of interest.
